# Ribosomal DNA localization on *Lathyrus* species chromosomes by FISH

**DOI:** 10.1186/s43141-020-00075-1

**Published:** 2020-10-20

**Authors:** Hoda B. M. Ali, Samira A. Osman

**Affiliations:** Genetics and Cytology Department, Genetic Engineering and Biotechnology Research Division, National Research Centre, Giza, P.O. 12622 Egypt

**Keywords:** *Lathyrus*, rRNA genes, FISH

## Abstract

**Background:**

Fluorescence *In Situ* Hybridization (FISH) played an essential role to locate the ribosomal RNA genes on the chromosomes that offered a new tool to study the chromosome structure and evolution in plant. The 45S and 5S rRNA genes are independent and localized at one or more loci per the chromosome complement, their positions along chromosomes offer useful markers for chromosome discriminations. In the current study FISH has been performed to locate 45S and 5S rRNA genes on the chromosomes of nine *Lathyrus* species belong to five different sections, all have chromosome number 2n=14, *Lathyrus gorgoni* Parl, *Lathyrus hirsutus* L., *Lathyrus amphicarpos* L., *Lathyrus odoratus* L., *Lathyrus sphaericus* Retz, *Lathyrus incospicuus* L, *Lathyrus paranensis* Burkart, *Lathyrus nissolia* L., and *Lathyrus articulates* L.

**Results:**

The revealed loci of 45S and 5S rDNA by FISH on metaphase chromosomes of the examined species were as follow: all of the studied species have one 45S rDNA locus and one 5S rDNA locus except *L. odoratus* L., *L. amphicarpos* L. and *L. sphaericus* Retz L. have two loci of 5S rDNA. Three out of the nine examined species have the loci of 45S and 5S rRNA genes on the opposite arms of the same chromosome (*L. nissolia* L., *L. amphicarpos* L., and *L. incospicuus* L.), while *L. hirsutus* L. has both loci on the same chromosome arm. The other five species showed the loci of the two types of rDNA on different chromosomes.

**Conclusion:**

The detected 5S and 45S rDNA loci in *Lathyrus* could be used as chromosomal markers to discriminate the chromosome pairs of the examined species. FISH could discriminate only one chromosome pair out of the seven pairs in three species, in *L. hirsutus* L., *L. nissolia* L. and *L. incospicuus* L., and two chromosome pairs in five species, in *L. paranensis* Burkart, *L. odoratus* L., *L. amphicarpos* L., *L. gorgoni* Parl. and *L. articulatus* L., while it could discriminate three chromosome pairs in *L. sphaericus* Retz. these results could contribute into the physical genome mapping of *Lathyrus* species and the evolution of rDNA patterns by FISH in the coming studies in future.

## Background

Genus *Lathyrus* L. is one of the many genera of the family Leguminosae, genus *Lathyrus* includes as many as 187 species and sup-species, most of them are annual species and a few are perennial species [1], According to [2], 13 intrageneric sections have been recognized in the genus *Lathyrus* out of them section *Lathyrus*, which comprises about 30 species. *Lathyrus* species dispersed all over temperate regions of the northern hemisphere and spreads into tropical East Africa and South America. The main center of genus *Lathyrus* diversity is in the Mediterranean and Irano-Turanian regions, there are fewer centers in North and South America [2]. Members of the *Lathyrus* genus are used in agriculture, some as fodder crop (*L. hirsutus* and *L. palustris*), and human nutrition (*L. sativus*), some are grown as ornamentals, for instance, *L*. *odoratus* [3, 4]. Grass pea (*L. sativus* L.) is the most investigated *Lathyrus* species due to its importance as human consumption plant, it has survived and spread over three continents and considered one of the most resistant plant species to environmental stress and climate changes [5–7].

Cytologically the basic chromosome number in genus *Lathyrus* is x = 7, and most of the species are diploid, or allopolyploids and a few are natural autopolyploids [8–11]. Despite the stability in the chromosome number and similarity in chromosome morphology, considerable variations in chromosome size which are associated with a fourfold variation in 2C nuclear DNA amount (6.9–29.2 pg/2C) have played an important role in the evolution of *Lathyrus* species [12–14].

Fluorescence *In Situ* Hybridization (FISH) has made a revolution in the cytogenetic because it could explain in more detail many questions related to karyotype diversity, organization and evolution of not only individual chromosomes but also entire genomes [15–22]. Many investigations have used FISH against various taxa to distinguish specific chromosomes, or to identify individual genome in the allopolyploidy species [23–28]. In plants, FISH has been used to localize a single copy gene on its position on a specific chromosome [29–31], by using bacterial artificial chromosome clones (BACs) clones was useful to paint specific chromosome [32–35], to reveal chromosomal inversion [36], or a translocation between the chromosomes of the different genomes in the allopolyploid hybrids [37], it was useful in designing species-specific DNA sequences (probes) to be tested on the related species, arising in comparative cytogenetic mapping among these species [38–41].

FISH investigations using rDNA sequences as probes remain the most common, probably because the sequences are highly conserved and occur as tandemly arranged repetitive copies that vary greatly in their number among the species. Two types of rDNA (45S and 5S rDNA) are present in eukaryotes and physically they are separated from each other [42–45]. Many cytological studies have been performed on *Lathyrus* species to compare the karyotype of different species [10, 11, 46–48], or to find out the chromosome banding patterns [49–52], at the cytogenetical level FISH was very useful to localize 45S and 5S rRNA genes on different *Lathyrus* species [51, 53–58]. The main target of this investigation is to locate 45S and 5S rDNA on nine *Lathyrus* species belong to five sections by FISH.

## Methods

### Plant material

All of the examined species have somatic chromosome number of 2n=14 chromosomes and belong to five sections. *L. gorgoni* Parl (accession no. LAT. 101), *L. hirsutus* L. (accession no. LAT. 167), *L. amphicarpos* L. (accession no. LAT. 137), and *L. odoratus* L. (accession no. LAT. 35) belong to section *Lathyrus*, *L. sphaericus* Retz (accession no. LAT. 137) and *L. incospicuus* L. (accession no. LAT. 164) belong to section *Linrearicarpus*. The other three species belong to different sections, *L. paranensis* Burkart (accession no. LAT.169) belongs to section *Notolathyrus*, while *L. nissolia* L. (accession no. LAT.168) belongs to section *Nissolia* and *L. articulatus* L. (accession no. LAT. 117) belongs to section *Clymenum* (Table 1). The nine *Lathyrus* species were obtained from the germplasm collection of the Institute of Plant Genetics and Crop Plant Research (IPK), Gatersleben, Germany.

**Table 1:** The sections of the studied species and the chromosome no. and the arm which display 5S rDNA or 45S rDNA

Species	Section	rDNA loci on chromosomes	
		5S rDNA	45S rDNA
*L. paranensis* Burkart	Notolathyrus	3*p*	4*q*
*L. nissolia* L*.*	Nissolia	1*p*	1*q*
*L. odoratus* L.	Lathyrus	3p**	4*q*
*L. hirsutus* L.	Lathyrus	3*p*	3*p*
*L. amphicarpos* L.	Lathyrus	1*p* + 2*p*	1*q*
*L. gorgoni* Parl.	Lathyrus	5*q*	3*q*
*L. articulatus* L.	Clymenum	1*p*	4*q*
*L. sphaericus* Retz	Linrearicarpus	2*p* + 3*p*	4*q*
*L. incospicuus* L.	Linrearicarpus	1*p*	1*q*

*p* Short arm *q* Long arm ** Two loci on the same chromosome arm

### Chromosome preparation:

Chromosome preparations from root tips and FISH were done according to [59] with minor modifications. Seeds were sown on two layers of moistened filter paper in a Petri dish and kept in the dark at 25°C for two days. The young germinated root tips were cut and treated with 0.02% aqueous 8-hydroxyquinoline for 3 h at 15°C and then washed several times with sterile water before fixation in freshly prepared Chloroform- acetic acid - ethanol (6:3:1) then in acetic acid - ethanol (1:3) and stored in ethanol 70%.The roots were washed in distilled water two times / 5 min and in citrate buffer (10 mM Na Citrate, pH 4.8) for 5 min, then softened in an enzyme mix [2% cellulase, and 1% pectinase (Sigma)] dissolved in the citrate buffer in an incubator at 37°C for 2 h. The root tips were washed again in citrate buffer and squashed on the slides in a drop of 45% acetic acid. The preparations were staged with a phase contrast microscope to select the slides with good separated chromosomes for further FISH experiment, then frozen on dry ice, washed with the fixation buffer and air-dried after removal of coverslips.

### Fluorescence *In Situ* Hybridization:

The *A. thaliana* BAC clone T15P10 (AF167571) bearing the 45S rDNA sequence was labelled with digoxigenin by nick translation, and the 5S rDNA probe was amplified from genomic DNA of *A. thaliana* and labelled with biotin by PCR with primers specific for the coding region [60]. The biotinylated 5S rDNA was detected by avidin~Texas Red (Vector Laboratories) and amplified by biotinylated goat anti-avidin (Vector Laboratories) and avidin~Texas Red. Digoxigenin-labelled probes were detected by mouse anti-digoxigenin (Jackson ImmunoResearch Laboratories) and goat anti-mouse antibodies conjugated with Alexa 488 (Molecular Probes). The chromosomes were counterstained with DAPI (2 μg/ml). The images were captured with a Zeiss Axioplan 2 epifluorescence microscope equipped with a Spot 2e CCD camera. Images were pseudo-coloured and merged using Adobe Photoshop CS software (Adobe). The karyotypes of the studied species have been done manually from the images which have been taken by the epifluorescence microscope. The available chromosome images were magnified in Adobe Photoshop CS software (Adobe) to enlarge the image to the size in which the difference in chromosome size could be identified, then each chromosome was copied and pasted separately and arranging the number of these chromosomes according to their decreasing in the size, taking in account the homologous chromosomes which bear the 45S and 5S rRNA genes.

## Results

FISH has been performed to locate 45S and 5S rRNA genes on the chromosomes of nine *Lathyrus* species all have 2n=14, and belong to five sections.

**Fig. 1:**
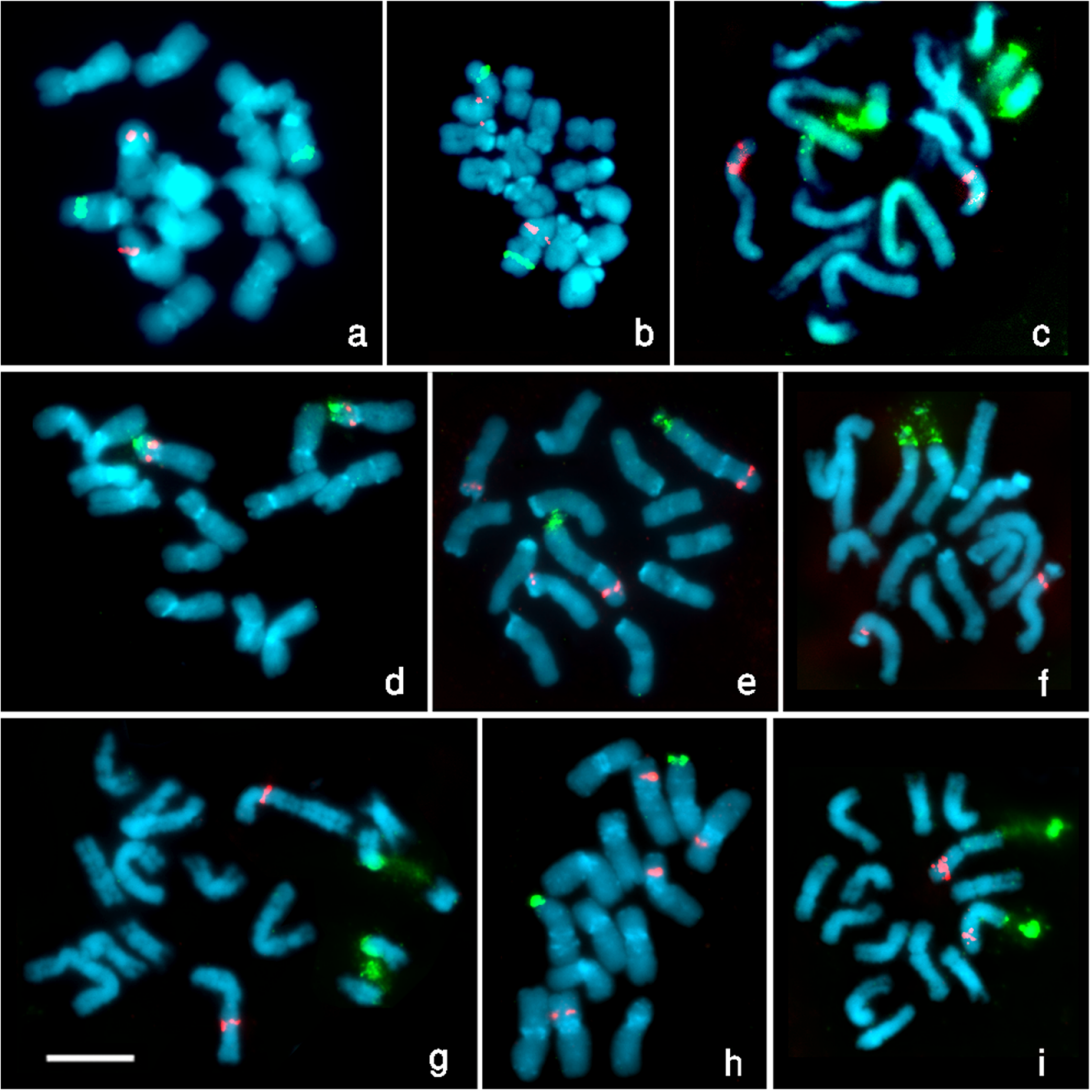
Mitotic metaphase chromosomes of nine *Lathyrus* species after FISH with rDNA probes, 45S rDNA probe was detected by FITC (green signals), and 5S rDNA probe by Texas red (red signals). The chromosomes were counterstained with DAPI. Bar = 5μ. **a**) *L. paranensis* Burkart **b**) L. nissolia L. **c**) L. odoratus L. **d**) *L. hirsutus* L. **e**) *L. amphicarpos* L. **f**) *L. gorgoni* Parl. **g**) *L. articulatus* L. **h**) *L. sphaericus* Retz **i**) *L. incospicuus* L.

**Fig. 2:**
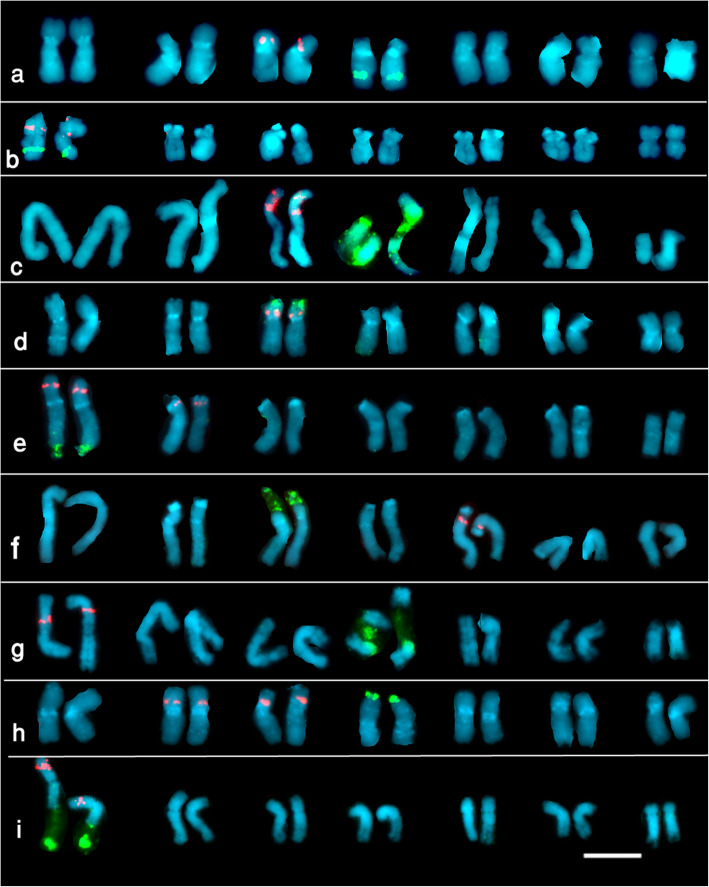
The Karyotypes of the examined *Lathyrus* species with 5S rDNA (in red) and 45S rDNA (in green) Bar = 5μ. **a**) *L. paranensis* Burkart **b**) *L. nissolia* L. **c**) *L. odoratus* L. **d**) *L. hirsutus* L. **e**) *L. amphicarpos* L. **f**) *L. gorgoni* Parl. **g**) *L. articulatus* L. **h**) *L. sphaericus* Retz **i**) *L. incospicuus* L.

The loci of 45S (in green) and 5S rDNA (in red) probes as revealed by double-target FISH on metaphase chromosomes preparations of the nine examined species have been shown in Fig. 1, and their karyotypes are shown in Fig. 2, and Table 1 summarizes the chromosome number and arm which display the 5S and 45S rDNA loci.

The detected 45S and 5S rRNA gene loci using double-FISH experiment on the metaphase chromosomes of each of studied species as follows:

***L. paranensis*** Burkart (Fig. 1a) belongs to section *Notolathyrus,* showed one interstitial 5S rRNA gene locus on the short arm of the chromosome no. 3 and one 45S rRNA gene locus on the long arm of chromosome no. 4.

***L. nissolia*** L. (Fig. 1b) belongs to section *Nissolia*, exhibited one interstitial locus of 5S rRNA and one 45S rRNA gene locus on the opposite arms of chromosome no. 1.

***L. odoratus*** L. belong to section *Lathyrus*, has one stretched interstitial 45S rRNA gene site on chromosome no. 4, which is distinguished with a big satellite, while two 5Sr RNA gene loci were observed in this species, one interstitial and one distal 5S rDNA on the short arm of chromosome pair no. 3 (Fig.1c).

***L. hirsutus*** L. belongs to section *Lathyrus* too, exhibited a single terminal 45S rDNA site, and one proximal 5S rDNA site located on the short arm of chromosome pair no. 3 (Fig.1d).

***L. amphicarpos*** L. the third studied species belongs to section *Lathyrus*, it has a big terminal 45Sr RNA gene locus on the long arm of the largest chromosome and interstitial 5Sr RNA gene locus on the short arm of the same chromosome (no. 1), in addition to another distal 5Sr RNA gene locus on the short arm of chromosome no. 2 (Fig. 1e).

***L. gorgoni*** Parl is the fourth studied species belongs to section *Lathyrus*, it was characterized by having one large stretched interstitial 5S rDNA site on the short arm of middle-sized chromosome pair (no.5), while the 45S rDNA site was at a distal position on the short arm of large chromosome pair no. 3 (Fig.1f).

***L. articulatus*** L. belongs to section *Clymenum*, it also exhibited one large stretched proximal 45S rDNA site on the long arm of chromosome pair no. 4, whereas the 5S rDNA site was located on the middle of the short arm of chromosome no. 1 (Fig. 1g).

***L. sphaericus*** Retz belongs to section *Linrearicarpus*. it was characterized by having two proximal 5S rRNA gene loci on the short arm of chromosomes no. 2 and no. 3, and one big terminal 45Sr RNA gene locus on the long arm of chromosome no. 4 (Fig. 1h)

***L. incospicuus*** L. belongs to section *Linrearicarpus* too, this species was characterized by having one distal 5S rRNA gene locus on the short arm of longest arm chromosome (no. 1), and exhibited one large stretched distal 45S rDNA site on the long arm of the same chromosome pair as well (Fig. 1i).

## Discussion:

Genus *Lathyrus* includes 187 species and sup-species, some of which have economic importance as food, fodder, or ornamental crops. *Lathyrus* species are distributed in the regions of the Northern Hemisphere and outspread into tropical South America East Africa [1, 16]. Most *Lathyrus* species are diploid (2*n* = 14). [2] classified genus *Lathyrus* depending on morphological traits into 13 intrageneric sections. Section *Nissolia* is monotypic has only one species *L. nissolia*. While section *Aphaca* is ditypic has two species *L. aphaca* and *L. stenolobus*, and section *Lathyrus* comprises about 30 species. Despite the stability in chromosome number, many investigations mentioned a variation in chromosome features, e.g. size, centromere position, and the size, number and position of secondary constrictions [11, 48, 52, 61, 62].

*Lathyrus* species show uniform chromosome morphology which is reflected in a homogeneous karyotype arrangement [9, 49]. Nevertheless, others have observed interspecific karyotype variations allowing species identifications [53, 61]. Such divergences have been also detected at the infraspecific level, especially in the extensively studied *L. odoratus* L. and *L. sativus* L. [53, 63, 64]. Many cytological studies have been performed to find out the chromosome banding patterns and karyotypes and /or ideograms of some *Lathyrus* species including *L. odoratus*, *L. articulatus* and *L. incospicuus* L. [49–52, 56], in previous investigation, the chromosome measurements, karyotype and chromosome banding patterns of six out of the nine studied species (*L. gorgoni* Parl, *L. hirsutus* L., *L. amphicarpos* L., *L. sphaericus* Retz, *L. paranensis* Burkart, and *L. nissolia* L.) have been studied by [52]. At the cytogenetical level, FISH was very useful to localize 45S and 5S rRNA genes on other *Lathyrus* species as well [51, 53–58].

Despite of few literatures stated the localization of rRNA gene loci by FISH in three of the examined species (*L. odoratus* L, *L. paranensis* Burkart and *L. hirsutus* L.), no previous FISH investigations have been found on the other six species. In *L. odoratus*, significate differences in its karyotype have been reported often to differ in the position and number of secondary constrictions, in the current study it was observed that *L. odoratus* (accession no. LAT. 35 ) has only one stretched interstitial 45S rRNA gene site on chromosome no. 4, which is distinguished with a big satellite. In the study of [63] they described cultivars with up to eight secondary constrictions, whereas [64] reported some with none at all. In the study of [53], silver staining (binds to the NOR, the nucleoli, and sometimes shows a tendency to bind chromosome centromere in some taxa, and dependent on the transcription rRNA genes) and *in situ* hybridization (independent of transcription and may also detect non-functional rDNA sites) were used to identify the nucleolar organizer regions (NORs) among six *Lathyrus* species, they noticed that *L. odoratus* and *L. hirsutus* were very similar with a large and a small sub-metacentric pair and five pairs of acrocentrics, and in both species the largest of the acrocentrics had a secondary constriction very close to the telomere of the short arm, in the same investigation they mentioned that *L. hirsutus* had a single pair of silver positive terminal spots. In *L. odoratus*, there was staining at or near the centromere in addition to staining at the secondary constrictions, but it was clear that the cells of *L. odoratus* have two pairs of terminal silver positive regions at the ends of two of the largest acrocentric pairs, and in the same study, the rDNA loci represented by FISH reflected the same number of signals in these two species.

The current study is in agreement with the result of [53] only with regard to *L. hirsutus* L., where it exhibited a single terminal 45S rDNA locus on the short arm of chromosome pair no. 3 and one proximal 5S rDNA site located in the middle of the same arm in this study, but with regard to *L. odoratus* L. disagrees. [46] used Silver staining as well to recognize the nucleolar organizer regions (NORs) in *L. odoratus* and they mentioned that there were four-terminal NORs on the short arms of pairs 4 and 5 with active ribosomal genes. However, *L. odoratus* L. bears microsatellites in two pairs of chromosomes (nos. 3 and 5) via the karyotype analysis by [11, 57] studied 14 species of *Lathyrus* (included *L. odoratus* L. and *L. paranensis* Burkart) by double FISH to determine the 45S and the 5S rDNA loci in addition to the CMA and DAPI banding patterns, they analyzed too the karyotypes in relation to geographic and climatic changes. In their investigation, they detected two loci of 45S rDNA on the short arm of chromosomes no. 4 and no. 5 in *L. odoratus* L., and they stated in *L. paranensis* one 5S rDNA locus and one 45S rDNA locus on separate chromosomes, which in agreement with the obtained result in the present study with regard to *L. paranensis* L., but disagrees with regard to *L. odoratus* L.

Nuclear DNA content may vary from 6.9–29.2 pg/2C [12–14] measured the nuclear DNA content (1C) in many plant genera, among them the genus *Lathyrus*, according to their measurements, there was no correlation between the genome size and the number or position of rDNA loci, nevertheless depending on the DNA content measurements (pg /1C) within section *Lathyrus* they recorded that *L. amphicarpos* L. and *L. gorgoni* Parl. were closely related (4.80, 5.80 pg/1C, respectively), and *L. hirsutus* L. (10.00 pg/1C) and *L. odoratus* L. (7.80 pg/1C) relatively similar too. In a former study [65] the DNA contents of different *Lathyrus* species have been measured by flow cytometry, among them *L. gorgoni* Parl (11.5 pg/2C), *L. hirsutus* L. (12.7 pg/2C) and *L. odoratus* L. (14.3 pg/2C), and the gradually increasing in the genome size in these three species is in agreement with their relationships as revealed by FISH in the current investigation.

The differences in genome size generally in plants and correspondingly in *Lathyrus* could be attributed first to the variations in the chromosome complements size and to the non-coding elements such as transposable elements, pseudogenes, and other repetitive sequences throughout the chromosome structure. The obtained banding patterns from many investigations supported the non-randomness of genomic change in *Lathyrus* as well, because the species mostly have uniform karyotypes and their banding patterns with similar quantity and base composition [11, 50]. Therefore, it is better to focus on the role of rDNA as repetitive sequences, rather than as coding gene loci and number [66] stated a slightly positive correlation between genome size and rDNA copy number in a restricted number of the eukaryotic test groups, and in some *Lathyrus* species by [51]. On the other hand [67] claimed against the existence of a precise relationship between the two parameters. The investigations of [68, 69] within the same taxonomic groups were also unsuccessful to reach this consensus, which in agreement with the obtained result of this study. The investigations of [70–74] informed that there is no correlation between rDNA copy number and genome size, and this correlation still a mystery because both characters are dynamic and are subjected for some changing during short periods of time. The chromosomal numbers, locations and structure of the 5S and 45S ribosomal DNAs (rDNA) in plants are nowadays available online [74, 75].

**Fig. 3:**
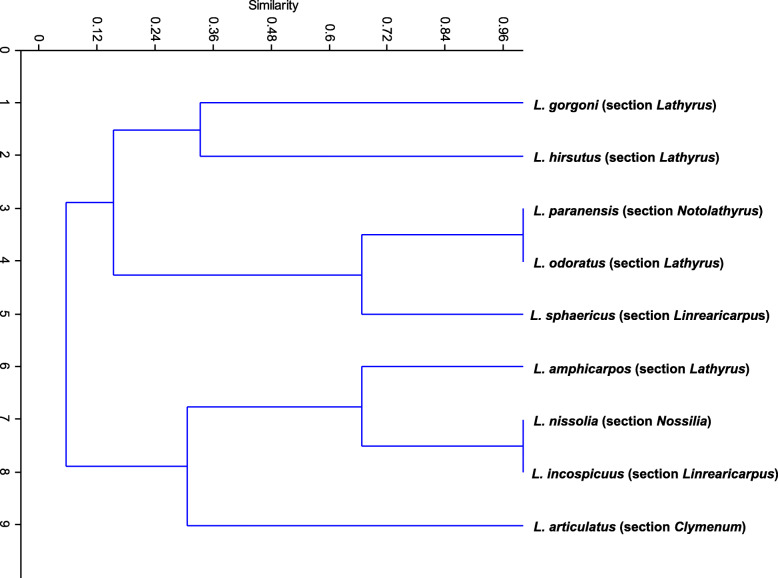
Dendrogram of the studied *Lathyrus* species based on the number and loci of 5S and 45S rDNA

FISH results of the investigated species in this study were summarized in Table 1, and Fig. 3. shows the dendrogram (UPGMA, suing Jaccard’s coefficient) which reflects the relationships among the studied species by Past program (Paleontological statistics software package for education and data analysis, [76])**,** which showed that the conventional plant taxonomy at the section level in the studied *Lathyrus* species is partially reflected by the number and loci of both rRNA genes. This is in agreement with the study of [77] based on amplified fragment length polymorphism (AFLP), where they found that *L. gorgoni* and *L. hirsutus* (section *Lathyrus*) grouped in same cluster, whereas *L. articulatus* (section *Clymenum*) was related to *L. inconspicuous* (section *Linearicarpus*), while *L. inconspicuous* and *L. sphaericus* (section *Linearicarpus*) were placed on distinct branches, and the results of [78] based on the internal transcribed spacer plus 5.8S-coding region of nuclear ribosomal DNA and cpDNA sequence data supported this. These relationships were partially reflected by the investigation of [79], who studied the relationships of different sections of genus *Lathyrus* depending on the cpDNA restriction site, and their obtained phylogenetic suggestions were used to test some species of genus *Lathyrus*, including *L. hirsutus* L. and *L. odoratus* L. which have been grouped under the same clade, while *L. gorgoni* Parl and *L. amphicarpos* L., under another clade, and *L. nissolia* L with *L. sphaericus* Retz in separate clade, whereas *L. paranensis* Burkart was found to be distantly related.

Double-target FISH by utilizing 5S and 45S rDNA loci as probes in the present study was helpful to discriminate the chromosomes of each of the nine studied species, which possibly could be used as chromosome markers. FISH could discriminate only one chromosome pair out of the seven pairs in three species, in *L. hirsutus* L., *L. nissolia* L. and *L. incospicuus*, and two chromosome pairs in five species, in *L. paranensis* Burkart, *L. odoratus* L., *L. amphicarpos* L., *L. gorgoni* Parl. and *L. articulatus* L., while it could discriminate three chromosome pairs in *L. sphaericus* Retz. these results could contribute into the physical genome mapping of *Lathyrus* species and the evolution of rDNA patterns by FISH in the coming studies in future.

## Conclusion:

Physical mapping of 5S and 45S rDNA loci by FISH on the chromosomes of the nine *Lathyrus* species could be used as chromosomal markers to discriminate the chromosome pairs of each of the examined species. FISH could discriminate only one chromosome pair out of the seven pairs in three species, in *L. hirsutus* L., *L. nissolia* L. and *L. incospicuus*, and two chromosome pairs in five species, in *L. paranensis* Burkart, *L. odoratus* L., *L. amphicarpos* L., *L. gorgoni* Parl. and *L. articulatus* L., while it could discriminate three chromosome pairs in *L. sphaericus* Retz. The conventional taxonomy at the section level in the studied *Lathyrus* species could not be proved by the number or loci of rRNA genes.

## Data Availability

Authors declare that all generated and analyzed data are included in the article. All plant materials (different *Lathyrus* species seeds) were identified and collected in Institute of Plant Genetics and Crop Plant Research (IPK), Gatersleben, Germany.

## Abbreviations

FISH: Fluorescence in situ Hybridization
NORs: nucleolar organizing regions
DAPI: 4′,6-Diamidin-2-phenylindol
rDNA: Ribosomal DNA
rRNA: Ribosomal RNA
cpDNA: chloroplast DNA
AFLP: Amplified Fragment Length Polymorphism
